# Multi-Target Actions of Acridones from *Atalantia monophylla* towards Alzheimer’s Pathogenesis and Their Pharmacokinetic Properties

**DOI:** 10.3390/ph14090888

**Published:** 2021-08-31

**Authors:** Pitchayakarn Takomthong, Pornthip Waiwut, Chavi Yenjai, Aonnicha Sombatsri, Prasert Reubroycharoen, Luo Lei, Ren Lai, Suchada Chaiwiwatrakul, Chantana Boonyarat

**Affiliations:** 1 Faculty of Pharmaceutical Sciences, Khon Kaen University, Khon Kaen 40002, Thailand; ppitcha.t@gmail.com; 2 Faculty of Pharmaceutical Sciences, Ubon Ratchathani University, Ubon Ratchathani 34190, Thailand; pwaiwut79@yahoo.com; 3 Faculty of Sciences, Khon Kaen University, Khon Kaen 40002, Thailand; chayen@kku.ac.th (C.Y.); aonnicha_s@hotmail.com (A.S.); 4 Center of Excellence in Catalysis for Bioenergy and Renewable Chemicals (CBRC), Department of Chemical Technology, Faculty of Science, Chulalongkorn University, Bangkok 10330, Thailand; prasert.r@chula.ac.th; 5 Kunming Institute of Zoology, the Chinese Academy of Sciences, Kunming 650223, China; luolei@mail.kiz.ac.cn (L.L.); rlai@mail.kiz.ac.cn (R.L.); 6 Faculty of Humanity and Social Sciences, Ubon Ratchathani Rajabhat University, Ubon Ratchathani 34000, Thailand; suchadachai65@gmail.com

**Keywords:** multi-target drugs, molecular docking, enzyme kinetic analysis, structure activity relationship, ADMET profiles

## Abstract

Ten acridones isolated from *Atalantia monophylla* were evaluated for effects on Alzheimer’s disease pathogenesis including antioxidant effects, acetylcholinesterase (AChE) inhibition, prevention of beta-amyloid (Aβ) aggregation and neuroprotection. To understand the mechanism, the type of AChE inhibition was investigated in vitro and binding interactions between acridones and AChE or Aβ were explored in silico. Drug-likeness and ADMET parameters were predicted in silico using SwissADME and pKCSM programs, respectively. All acridones showed favorable drug-likeness and possessed multifunctional activities targeting AChE function, Aβ aggregation and oxidation. All acridones inhibited AChE in a mixed-type manner and bound AChE at both catalytic anionic and peripheral anionic sites. In silico analysis showed that acridones interfered with Aβ aggregation by interacting at the central hydrophobic core, C-terminal hydrophobic region, and the key residues 41 and 42. Citrusinine II showed potent multifunctional action with the best ADMET profile and could alleviate neuronal cell damage induced by hydrogen peroxide and Aβ_1-42_ toxicity.

## 1. Introduction

Alzheimer’s disease (AD) is a complex neurodegenerative disease that has a slow and irreversible progression. AD symptoms are the result of brain structure changes in the hippocampus and cerebral cortex. The hippocampus is essential for learning and memory, which is susceptible to damage at early stages of AD, and the disturbance of cerebral cortex function affects orientation in space, language and memory [1]. The neuropathological features of AD include the accumulation of amyloid beta (Aβ) plaques and neurofibrillary tangles, the reduction of acetylcholine (ACh) level and oxidative stress, all leading to the loss of synapse and neuron function [2,3,4].

The formation of amyloid senile plaques is accepted to be a key feature of Alzheimer’s disease pathogenesis [5]. The major component of the senile plaques is Aβ peptides, which are derived from the proteolytic processing of β-amyloid precursor protein (APP). The most abundant Aβ peptides are made up of 40 and 42 amino acids [6,7]. Generally, Aβ peptides are thought to be unstructured in the monomer state, then they aggregate to form fibrils with an ordered cross-β-sheet pattern [8,9]. Several studies suggest that both the soluble oligomers and the mature fibrils are toxic forms [10,11]. Previous studies have found that backbone hydrogen bonds and salt bridges predominantly stabilize the anti-parallel intra-molecular β-strands of Aβ peptides, while hydrophobic interactions and the backbone of hydrogen bonds stabilize the parallel inter-molecular β-sheets [12,13,14,15,16,17,18]. Therefore, interference with the β-sheet and/or amyloid fibril formation could be considered as an approach to stopping or slowing the progression of AD. Recently, in June 2021, the first anti-Aβ aggregation drug, aducanumab, was approved by the FDA as an anti-Aβ antibody for AD treatment.

Several studies have suggested that the memory impairment in AD patients is related to the decline in ACh level in the cholinergic system. ACh is a major neurotransmitter in the brain located in the cortex, basal ganglia, and basal forebrain [19]. It is hydrolysed to acetate and choline by acetylcholinesterase (AChE), which is an important enzyme in cholinergic synapses. AChE inhibitors are therefore used for stabilizing ACh neurotransmitter levels in the synaptic cleft. The main role of AChE in cholinergic neurotransmission is to terminate ACh function at the post-synaptic membrane. The AChE enzyme contains a pocket with two binding sites, which include a catalytic ionic site (CAS) and a peripheral anionic site (PAS). The CAS is at the surface of the base of the gorge. It consists of two subsites including the esteratic site and the anionic site. The esteratic site has three key amino acids (Ser200, Glu327, and His440), which are called the catalytic triad, and they directly contribute to ACh hydrolysis. The anionic site is located in the middle gorge and consists of negatively charged amino acid residues (Trp84, Tyr130, Phe330 and Phe331). These amino acids are responsible for the molecular recognition of quaternary ammonium ligands and appropriately orientate ACh during the catalytic process. The hydrophobic amino acids (Tyr70, Asp72, Tyr121, Trp279, and Tyr334) located at the rim of the gorge referring to PAS act as a blockade to entry. During ACh hydrolysis, an incoming ACh molecule at the PAS blocks the entry of substrates and the exit of products from the active site [20,21]. Interestingly, the PAS region of AChE also participates in Aβ aggregation by accelerating the fibril aggregation process [22,23]. Recent studies reported that dual-binding AChE inhibitors targeting both CAS and PAS might not only attenuate memory impairments in AD patients by increasing ACh levels, but also provide additional benefit through delaying amyloid plaque accumulation [15,24,25,26].

Oxidative stress is also considered as an important factor contributing to the initiation and progression of AD [27,28]. However, the exact mechanism remains elusive. Oxidative injury in AD patients might result from mitochondrial abnormalities [29,30], altered antioxidant defenses [31] or β-amyloid-induced free radicals [32,33,34]. To combat oxidative stress in AD, treatment with antioxidants might be a promising strategy for slowing AD progression. Within the last few years, a number of antioxidants have been described that possibly benefit AD patients [35,36].

Currently, there are only six drugs approved for AD by the Food and Drug Administration (FDA) including donepezil, rivastigmine, galantamine, memantine, Namzaric (a combination of donepezil and memantine) and aducanumab. However, the current approved drugs can only provide partial symptomatic relief and limited improvement in daily living ability. These classical drugs act by modulating a single target, which might not be effective for this complicated disease. Thus, multi-targeted drugs could provide better effective treatments.

Tacrine, a member of the acridine class, was the first potent AChE inhibitor approved for AD by the FDA. Its mechanism of action involves several pathways including cholinergic, glutaminergic, and gabaergic pathways. However, it was withdrawn from the market due to serum aminotransferase elevation and acute liver injury [37,38,39]. Several AChE inhibitors have been developed based on the tacrine scaffold due to their potential multi-target action in AD. Acridone alkaloids, which have similar structure to tacrine, are composed of fused heterocyclic rings containing nitrogen at position 10 and a carbonyl group at position 9. Acridone is a unique alkaloid known to possess several biological activities including anti-cancer [40], anti-microbial [41], anti-pruritic [42] and anti-viral [43] activities. Moreover, some acridone derivatives have been shown to inhibit AChE activity [44]. Hence, acridone alkaloids might be a potential compound for AD treatment.

**Figure 1:**
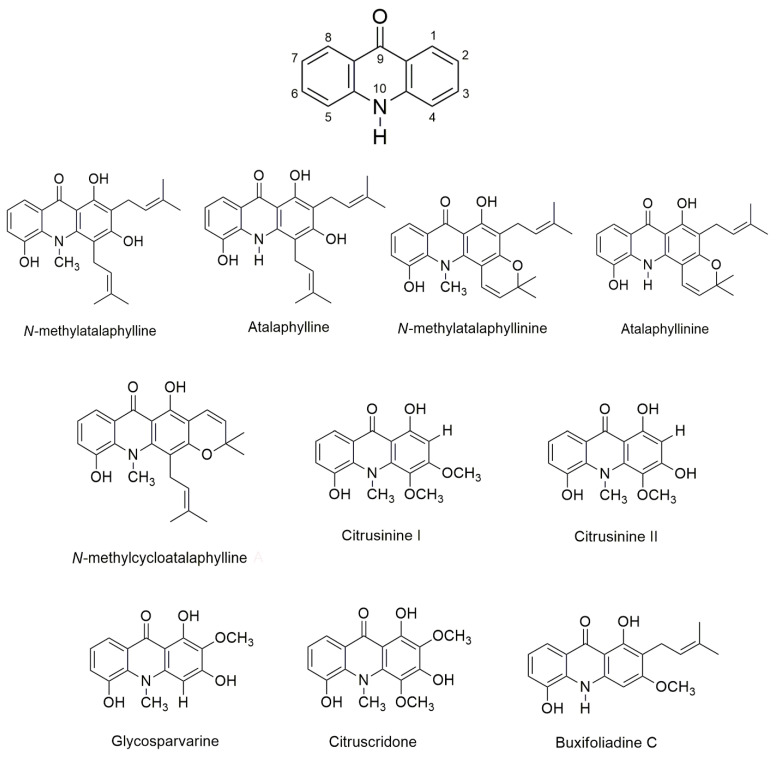
Structure of acridone alkaloids.

The aim of this study was to investigate the anti-AD activities of acridones previously isolated from *Atalantia monophylla* [45], namely *N*-methylatalaphylline, atalaphylline, *N*-methylatalaphyllinine, atalaphyllinine, *N*-methylcycloatalaphylline A, citrusinine I, citrusinine II, glycosparvarine, citruscridone, and buxifoliadine C (Figure 1). All ten acridones were evaluated for drug-likeness properties by in silico analysis (SwissADME web tool). The inhibition of AChE function and Aβ fibril aggregation was performed both in vitro and in silico (AutoDock program). Moreover, acridones were evaluated for their antioxidant and neuroprotective effects against H_2_O_2_ and Aβ_1-42_-induced cell damage. Finally, the pharmacokinetic properties of the acridone derivatives were studied in silico (pKCSM program).

## 2. Results and Discussion

### 2.1. Physicochemical Properties of Acridones

In the drug discovery process, it has been reported that 95% of drug candidate molecules fail in the development stages, and 50% of such failures are caused by unsatisfactory physicochemical and ADMET properties. To avoid this failure, test compounds should be filtered by their drug-likeness properties. Drug-likeness is a useful concept in virtual screening that improves the chance of chemical entities and avoids failure in drug development. Therefore, drug-likeness is a helpful tool to select appropriate chemical candidates. The assessment of drug-likeness properties is based on chemical structures and physicochemical properties. Thus, in this study, we used SwissADME webserver to estimate the physicochemical features. The results are represented in Table 1. All 10 tested acridones showed no violation of Lipinski’s rules. Their LogP values were within the range of 1.66 to 4.61. The molecular weights, number of hydrogen bond acceptors and number of hydrogen bond donors were within the accepted values of less than 500, 10 and 5, respectively [46]. Moreover, all the acridones followed the criteria of Veber’s rules with total polar surface area (TPSA) values and numbers of rotatable bonds within the range for oral availability [47]. Thus, all acridones showed favorable drug-likeness, which means they might be good drug candidates.

### 2.2. Antioxidant Activity

There is substantial evidence that free radical-induced oxidative damage may play a role in the pathogenesis of AD. Free radicals are reactive oxygen compounds that may attack and damage lipids, proteins, and DNA in the brains of AD patients. Therefore, scavenging free radicals might be a promising approach for slowing disease progression. In the present study, the antioxidant activity of the 10 acridones was evaluated by ABTS radical scavenging assay and trolox was used as the reference compound. The ability of the test compounds to scavenge ABTS radicals is represented as IC_50_, which is the test compound concentration that resulted in 50% inhibition of free radicals. The ABTS radical scavenging activity of acridones is summarized in Table 2. All acridone alkaloids exhibited moderate to good antioxidant activity with IC_50_ values ranging from 19.98 to 79.58 µM. Among all tested acridone alkaloids, citrusinine I, citrusinine II, glycosparvarine, buxifoliadine C and citruscridone possessed potent free radical stabilization capacity. Interestingly, they showed better activity than the reference compound, trolox.

The results also showed that there is a correlation between the number of hydroxyl groups and antioxidant activity. Citrusinine II and glycosparvarine, which contain 3 hydroxy groups on the acridone ring, showed the most potent radical scavenging action with IC_50_ values of 19.98 and 22.88 μM, respectively. When the hydroxy group at position 3 was substituted with a methoxy group, the antioxidant activity was decreased, as shown by the increased ABTS radical scavenging IC_50_ value of 33.77 μM for citrusinine I. The addition of bulky residues such as methoxy or prenyl groups at positions 2 or 4 on the acridone moiety also proved unfavorable to radical scavenging action. Citruscridone (IC_50_ = 31.69 μM) and N-methylatalaphylline (IC_50_ = 50.73 μM), bearing methoxy and prenyl groups, respectively, possessed weaker activity than citrusinine II (IC_50_ = 19.98 μM). These reductions in radical scavenging activity might result from steric hindrance by the bulky groups. Moreover, cyclization at positions 2–3 or 3–4 on the acridone alkaloid ring diminished the number of hydroxyl groups, which decreased the radical scavenging activity. N-methylcycloatalaphylline (IC_50_ = 79.58 μM) and N-methylatalaphyllinine (IC_50_ = 57.53 μM) showed less radical scavenging activity than N-methylatalaphylline (IC_50_ = 50.73 μM). From the structural activity relationship, it could be concluded that the number of hydroxyl groups on the acridone ring influenced the free radical scavenging ability. The hydroxyl group, an electron donor, enhances free radical scavenging by liberating electrons to free radicals. This is consistent with previous studies that reported correlations between the number of hydroxy groups on aromatic rings and the radical scavenging activity [48]. However, bulky substituents neighboring hydroxy groups might also attenuate radical scavenging activity by generating steric hindrances.

### 2.3. AChE Inhibition and Molecular Docking Studies

**Figure 2:**
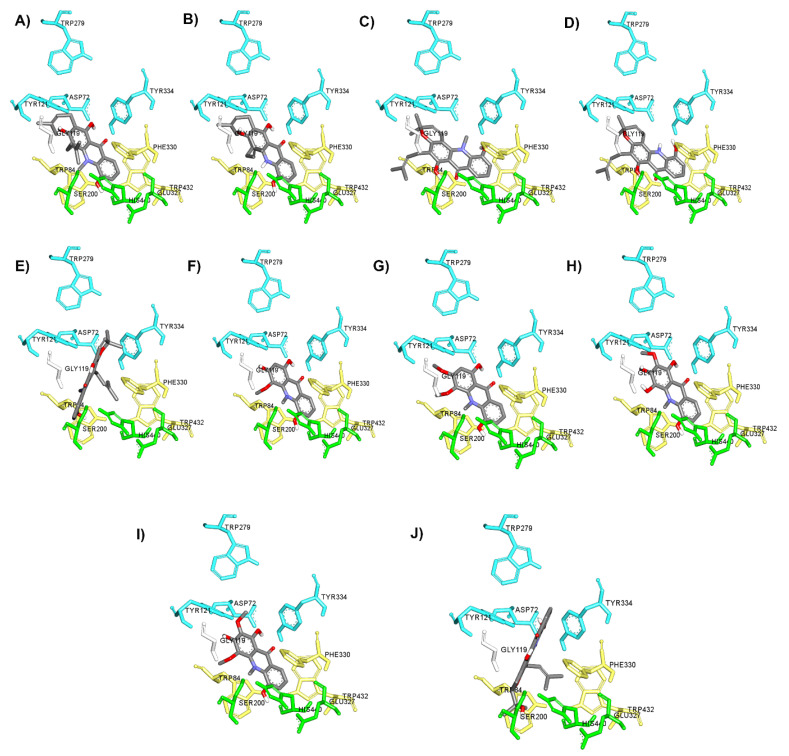
Binding orientation for interactions of acridone derivatives N-methylatalaphylline (**A**), atalaphylline (**B**), N-methylatalaphyllinine (**C**), atalaphyllinine (**D**), N-methylcycloatalaphylline A (**E**), citrusinine II (**F**), citrusinine I (**G**), glycosparvarine (**H**), citruscridone (**I**), and buxifoliadine C (**J**) docked into AChE. The binding sites are coded with different colors (catalytic triad; green, anionic site; yellow, peripheral site; blue).

**Figure 3:**
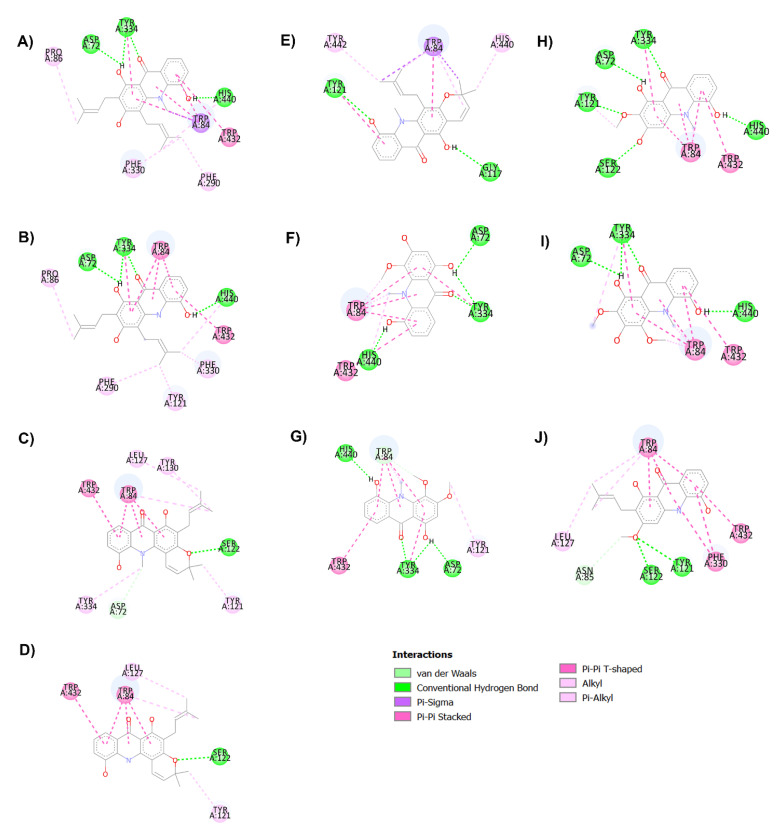
Binding interaction diagram of acridone derivatives N-methylatalaphylline (**A**), atalaphylline (**B**), N-methylatalaphyllinine (**C**), atalaphyllinine (**D**), N-methylcycloatalaphylline A (**E**), citrusinine II (**F**), citrusinine I (**G**), glycosparvarine (**H**), citruscridone (**I**), and buxifoliadine C (**J**) bound to AChE.

It is well-known that the cholinergic system plays a vital role in the regulation of the learning, cognitive and memory processes [49]. Evidence has suggested that a decline in the level of the neurotransmitter acetylcholine, which plays a role in learning and memory functions [50], appears to be a critical element in the development and progression of AD. Hydrolysis of ACh through AChE results in termination of cholinergic transmission; therefore, inhibition of AChE can serve as a therapeutic target for AD treatment. In the present study, all acridones were tested for their inhibition of AChE according to the method of Ellman and co-workers, using tacrine as a reference compound. The inhibitory activities of test and reference compounds are summarized in Table 2. The test compounds exhibited moderate to good activity against AChE with IC_50_ values ranging from 18.61 to 66.62 µM. Then, to further delineate the underlying mechanism, the binding interactions between acridones and AChE were investigated by using Autodock 4.2.6 and Discovery studio programs. The binding modes and interaction diagrams of all acridones bound to AChE are shown in Figure 2 and Figure 3. Interestingly, the docking simulations revealed that all acridone derivatives simultaneously occupied both the CAS and PAS of AChE (Figure 2). Acridone consists of two benzene rings fused together with a keto group and a nitrogen atom at positions 9 and 10, respectively, resulting in a planar structure. The binding orientation analysis revealed that the planar structure of the acridone ring that is present in all derivatives allows the establishment of a π–π stacking interaction with Trp84 and Trp432 in the AS, which plays a role in binding the quarternary ammonium of the substrate, ACh (Figure 3). Moreover, the presence of a hydroxy group at position 5 of the acridone moiety usually created a hydrogen bond with His440, a key amino acid of the catalytic triad in CAS, which is responsible for the hydrolysis of ACh. Furthermore, carbonyl groups at position 9 and hydroxyl groups at position 1 could form hydrogen bonds with key residues in the PAS, which is involved in acceleration of the Aβ aggregation process. Thus, the docking results confirmed that the majority of the acridone derivatives were located in the CAS and PAS active sites, thereby interfering with the hydrolysis of ACh and with Aβ aggregation.

Among all acridones, glycosparvarine exhibited the highest AChE inhibitory activity with an IC_50_ value of 18.61 μM. The core structure of glycosparvarine lays along with the middle gorge of the AChE active site by locating ring A in the CAS and ring B in the PAS regions (Figure 2H). Ring A interacted with Trp84 and Trp432 in the CAS via a π–π stacking interaction (Figure 3H). Ring B formed a π–π stacking interaction with Tyr 334 in the PAS. Glycosparvarine bound to AChE with five hydrogen bonds. The hydroxyl group at position 5 interacted with His440 in the CAS via a hydrogen bond. The carbonyl group at position 9, hydroxyl group at position 1 and methoxy group at position 2 formed hydrogen bonds with Tyr334, Asp72 and Tyr121, respectively, in the PAS, which is involved with the acceleration of the Aβ aggregation process. Moreover, the hydroxyl at position 3 formed a hydrogen bond with Ser122. The docking results indicate that glycosparvarine could interact with AChE through dual mode binding including both the CAS and PAS binding sites. Glycosparvarine was more potent than citruscridone, which has a methoxy group at position 4. The substitution of a methoxy group at this position has a steric hindrance that terminates the hydrogen bond interaction between the hydroxyl group at position 3 and Ser122, leading to decreased inhibitory potency (Figure 3H,I). Similarly, the presence of a prenyl group in position 4 of N-methylatalaphylline also decreased AChE inhibitory activity due to steric hindrance (Figure 3A). It is therefore suggested that the bulkiness of the substituents at position 4 had an effect on the inhibition of AChE activity via steric hindrance. In contrast, the change of a hydroxyl group at position 3 to a methoxy group resulted in an increase in activity. Citrusinine I (Figure 3G) showed more potent activity than citrusinine II (Figure 3F) due to the additional hydrophobic interaction of the methoxy group at position 3 with Tyr121 in the PAS region.

Regarding substituents at the 10th position of the acridone ring, the substitution of a methyl group resulted in an activity increase. N-methylatalaphylline (IC_50_ = 49.08 μM) and N-methylatalaphyllinine (IC_50_ = 54.04 μM) were slightly more potent than atalaphylline (IC_50_ = 58.33 μM) and atalaphyllinine (IC_50_ = 60.78 μM), respectively. The docking analysis indicated that the N–CH_3_ groups of N-methylatalaphylline (Figure 3A) and N-methylatalaphyllinine (Figure 3C) could form hydrophobic interactions with Trp84 and Tyr334, respectively. Thus, the presence of N–CH_3_ at position 10 would slightly improve the inhibitory effect by enhancing the hydrophobic interaction with key amino acids of AChE.

N-methylcycloatalaphylline A (IC_50_ = 66.62 μM) and N-methylatalaphyllinine (IC_50_ = 54.04 μM) possessed less potent activity than N-methylatalaphylline (IC_50_ = 49.08 μM). This decrease in activity could be attributed to the cyclization at positions 2–3 and 3–4, which may cause steric hindrance and interfere with the binding interaction. The docking analysis showed that the binding orientations of N-methylcycloatalaphylline A and N-methylatalaphyllinine were quite different from N-methylatalaphylline as their conformations were flipped within the active site, resulting in the deviation of the acridone ring from the catalytic triad (Figure 3C,E).

Taken together, these results suggest that all acridone derivatives could interact with AChE through dual mode binding. The acridone moiety plays a pivotal role in the inhibition of AChE activity by interacting with Trp84 and Trp432 in the middle gorge of AChE and the hydroxy group at position 5 could bind to His440 in CAS. Therefore, the acridones locate at the CAS of AChE, thereby interfering with the hydrolysis of ACh. Moreover, the acridones with substituents at positions 1, 2 and 3 could bind to the PAS via hydrogen bonds with Tyr334, Asp72 and Tyr121, to affect the Aβ aggregation process.

### 2.4. Kinetic Analysis of AChE Inhibition

**Figure 4:**
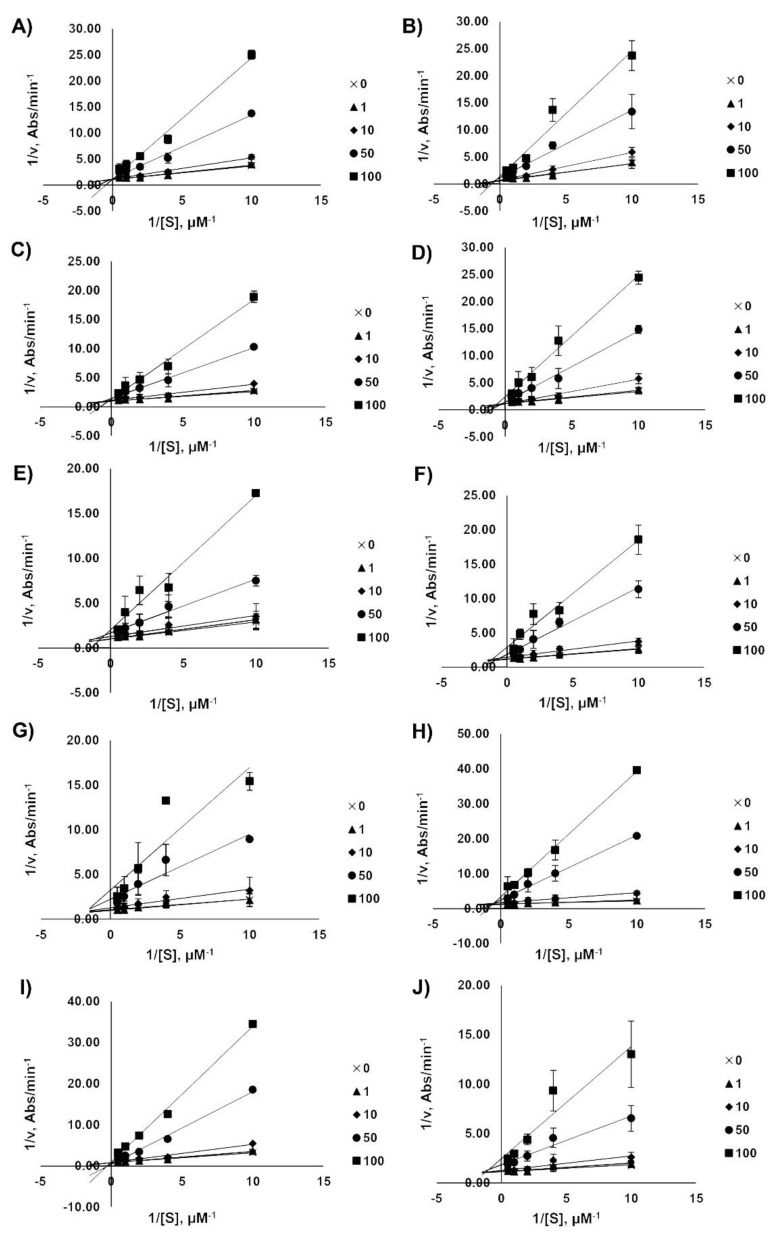
Lineweaver–Burk plots for the inhibition of AChE by acridones N-methylatalaphylline (**A**), atalaphylline (**B**), N-methylatalaphyllinine (**C**), atalaphyllinine (**D**), N-methylcycloatalaphylline A (**E**), citrusinine II (**F**), citrusinine I (**G**), glycosparvarine (**H**), citruscridone (**I**), and buxifoliadine C (**J**). The absorbance was measured by UV spectroscopy at the wavelength of 405 nm.

The enzyme inhibition mode was analyzed by a double-reciprocal Lineweaver–Burk plot as shown in Figure 4. The Lineweaver–Burk plots showed an increase in slope when the inhibitor concentrations were increased. These patterns indicated that all acridones showed mixed type inhibition against AChE, which were calculated from the Lineweaver–Burk model. Knowing the type of inhibition suggested that acridones could bind to both the CAS and PAS of AChE, but that they do not bind to the same binding site as the substrate. Among the tested acridones, the most active compound against AChE was glycosparvarine with an IC_50_ value of 18.61 ± 2.77 µM.

### 2.5. Anti-Aβ Aggregation Activity and Molecular Docking Studies

**Figure 5:**
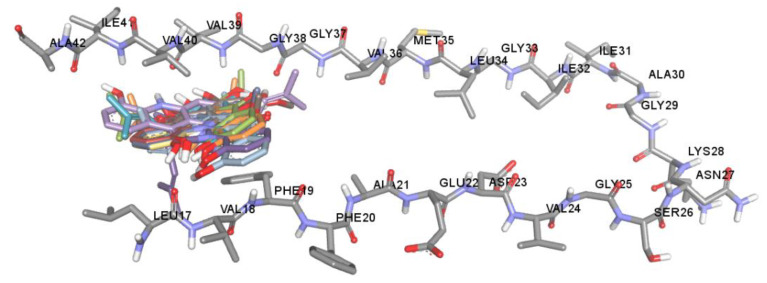
Superposition of docked orientations of all acridones on amyloid beta (PDB code: 2BEG).

**Figure 6:**
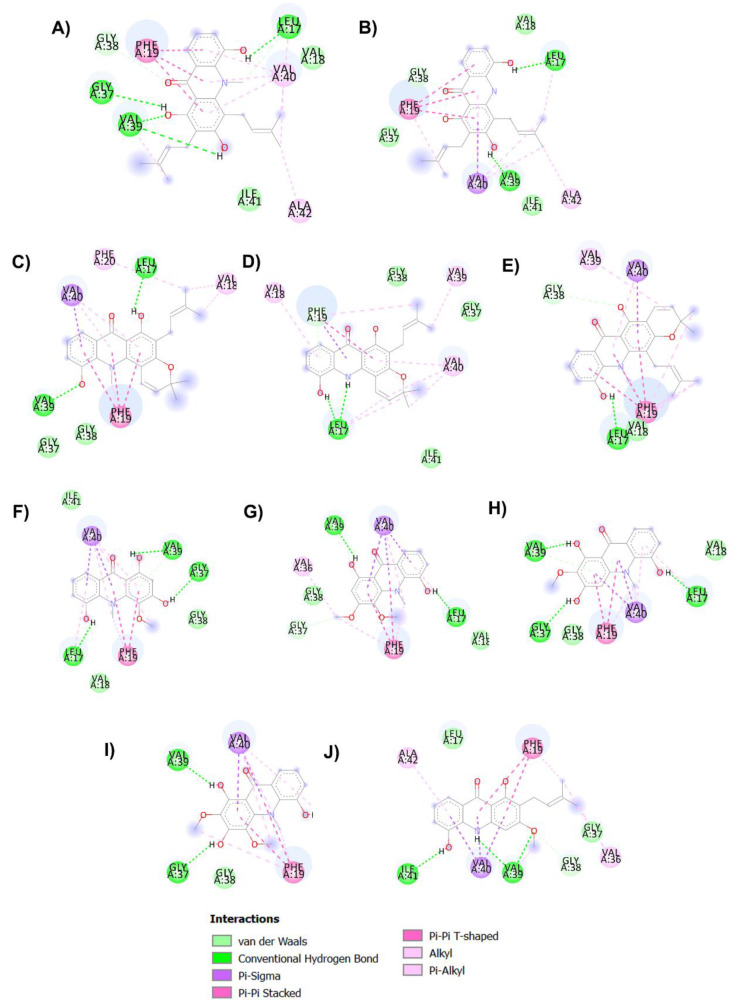
Binding interaction diagram of acridones *N*-methylatalaphylline (**A**), atalaphylline (**B**), *N*-methylatalaphyllinine (**C**), atalaphyllinine (**D**), *N*-methylcycloatalaphylline A (**E**), citrusinine II (**F**), citrusinine I (**G**), glycosparvarine (**H**), citruscridone (**I**), and buxifoliadine C (**J**) and Aβ_1-42_.

The key hallmark of AD pathogenesis is accumulation of toxic Aβ plaques in the brain. Therefore, preventing or reducing formation of Aβ plaques has been the primary goal of several therapeutic strategies under development or in clinical trials. The major components of senile plaques are Aβ_1-40_ and Aβ_1-42_ [6,7]. Aβ_1-42_ is known to have higher pathogenicity due to its higher tendency to self-assemble into fibrils [7]. Therefore, modulating Aβ_1-42_ aggregation could be an important therapeutic strategy for treatment of AD. Aβ_1-42_ fibrils are composed of a cross-β structure in which the N-termini (residues 1–17) are unstructured and residues 18–42 form the β-turn-β fold. The β-turn region is composed of two parallel, in-register β-sheets, which are from residues 17–21 and 29–35 [12,51,52]. To stabilize the turn formation, these two β-strands are connected by a loop region where a salt bridge is formed between Asp23 and Lys28 [12,51]. Furthermore, there are two molecular contacts that stabilize the β-sheet structure in which Phe19 packs against Gly38, and Ala42 contacts the side chain of Met35. The central hydrophobic region (residues 17–21) and the C-terminal hydrophobic segment (residues 39–42) have been identified as nucleation sites of Aβ aggregation. These regions act as a steric zipper, which leads to dimer formation and eventually larger aggregation. Interchain interactions are also observed along with hydrogen bonds between the backbone of residues Val18-Val39, Asp23-Leu34, Lys28-Val36, Glu22-Met35, Val36-Ile41, and Phe20-Gly37. Moreover, the addition of two amino acids, Ile41 and Ala42, has been shown to have a significant effect on disease progression. Ile41 is critical for the formation of paranuclei and Ala42 is necessary for assembly into larger oligomers [53,54]. Taken together, the central hydrophobic segment (residues 17–21), hydrophobic C-terminal (residues 39–42), and turn segment (residues 23–28) are critical regions of Aβ_1-42_ that may enhance conformational transition, initiate nucleation and promote fibril formation. Thus, compounds that have a tendency to interact with these binding regions tend to inhibit Aβ aggregation. Herein, the regulatory effect and mechanism of action of acridones on Aβ_1-42_ aggregation were studied using both in vitro and in silico procedures. All acridones were evaluated for their inhibitory action against self-mediated Aβ_1-42_ aggregation by using an in vitro thioflavin T (ThT) fluorometric assay and binding interactions between acridones and Aβ1-42 were investigated using the Autodock 4.2.6 program. The in vitro results demonstrated that all acridone alkaloids exhibited good Aβ_1-42_ aggregation inhibitory activity with IC_50_ values ranging from 4.79 to 8.81 µM, and no significant difference in inhibitory activity (Table 2). The molecular docking analysis confirmed that all acridones could bind with Aβ_1-42_ at the same binding region with slightly different conformations, as shown in Figure 5. The heterocyclic ring (acridine skeleton) of all acridones could form hydrophobic interactions with Phe19 and Val40 via π–π stacked and π-alkyl interactions, respectively (Figure 6). Moreover, the hydroxyl group at position 5 of acridones formed a hydrogen interaction with Leu17 in the central hydrophobic region. Thus, the core structure of all acridones located to the central hydrophobic core region of Aβ_1-42_, which is responsible for initiating nucleation and promoting fibril formation. Alteration of substituents on the acridine ring caused slight differences in inhibitory action. Substitution with hydroxyl groups at positions 1, 3 or 5 of the acridine ring enhanced hydrogen bonding with Gly37, Val39, and Leu17, while substitution with methoxy or prenyl groups at positions 2, 3 or 4 promoted hydrophobic interactions with Gly37, Gly38, Val39, Val40, Ile41 or Ala42 in the C-terminal hydrophobic region. These amino acids are key residues in the hydrogen bonding and hydrophobic interaction networks of inter- and intra-molecular interactions that help maintain the stability of fibrils. Moreover, Ile41 is the residue that plays a principal role in the formation of paranuclei, whereas Ala42 is necessary for further assembly into larger oligomers [53,54]. The in silico data indicated that acridones may interfere with fibril stabilization through interactions with the central hydrophobic core and C-terminal hydrophobic region, as well as the residues at positions 41 and 42, which are the key segments accounting for Aβ nucleation and fibrillation.

### 2.6. In Silico ADMET Properties of Acridones

The parameters for evaluating ADMET were estimated using pKCSM [55] and are presented in Table 3. The in silico investigation of ADMET revealed that human intestinal absorption of acridones ranged from 68.876% to 99.555%. Among all acridones, citruscridone showed the poorest absorption, which might be a direct effect of TPSA as increased TPSA diminishes membrane permeability [56]. The brain distribution of acridones was examined by blood brain barrier (BBB) permeability (LogBB). The BBB protects the brain by preventing the access of polar molecules to the brain. Interestingly, the tested acridones can readily cross the BBB because their Log BB values are above −1. However, citruscridone appears unable to penetrate the BBB according to its poor absorption and high TPSA value.

The metabolism, excretion, and toxicity of the acridones were also analysed. Cytochrome P450 is a superfamily of important detoxification enzymes in the body that are primarily located in the liver and the small intestine. They oxidize xenobiotics and play a role in their clearance. The two most prevalent P450 drug metabolising enzymes in the liver and small intestine are P450 3A4 and P450 2C9 [57]. The inhibition of the CYP2C9 and CYP3A4 isoforms of cytochrome P450 by the 10 acridones is shown in Table 3. Only citrusinine II and glycosparvarine did not inhibit both isoforms of cytochrome P450. Thus, these two compounds might not affect CYP450 enzyme activity, which suggests that they will not affect the metabolism and clearance of the various drug substrates of CYP2C9 and CYP3A4.

The kidney also plays an important role in drug elimination. The organic cation transporter 2 (OCT2) is a primary renal uptake transporter that plays a role in the disposition and clearance of organic cation drugs. Our compounds are not likely to be OCT2 substrates as they did not display drug–drug interactions to decrease the renal clearance of an OCT2 substrate. The renal total clearance was also calculated and is presented in Table 3. For toxicity, hepatotoxicity is the main concern because the tested acridone compounds all retain the same core structure as tacrine. Tacrine was abandoned as an AD treatment, despite being a potent AChE inhibitor, due to its hepatotoxicity [58]. Interestingly, only citrusinine II, glycosparvarine, and citruscridone showed no hepatotoxicity. In summary, glycosparvarine and citrusinine II showed the best ADMET profiles. However, this needs to be confirmed in in vitro and in vivo studies.

### 2.7. Neuroprotective Effects against H_2_O_2_ and Aβ_1-42_-Induced Cell Death

**Figure 7:**
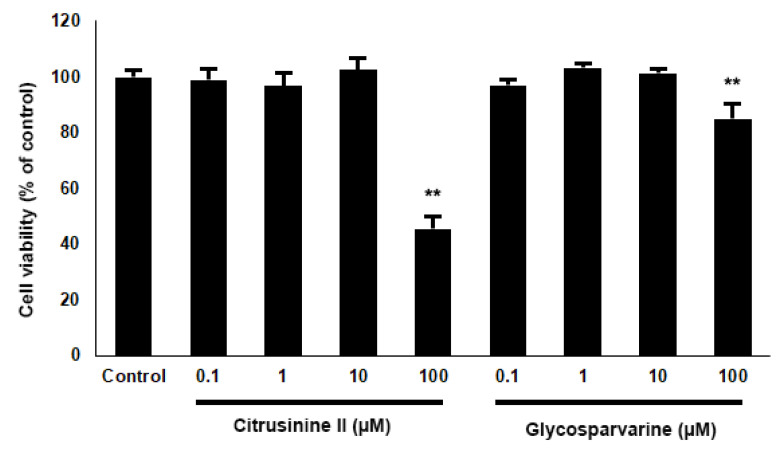
Effects of citrusinine II and glycosparvarine on cell viability of SH-SY5Y cells. The values are reported as mean ± SD (*n* = 4), One-way ANOVA followed by the Tukey test was used to compare between control and test inhibitors, ** *p* < 0.01.

Based on the in vitro biological activity and pharmacokinetic properties of the 10 acridones, citrusinine II and glycosparvarine were the most potent multifunctional acridones with the best ADMET profiles. Hence, citrusinine II and glycosparvarine were selected for further neuroprotective investigation. For cytotoxicity evaluation by MTT assay, both citrusinine II and glycosparvarine, at concentrations of 0.1–10 µM, showed no effect on viability of SH-SY5Y neuroblastoma cells after 24 h incubation as shown in Figure 7. Thus, the acridones were investigated for neuroprotective effects against H_2_O_2_ and Aβ_1-42_ induced toxicity in human SH-SY5Y neuroblastoma cells at the non-toxic concentration of 10 µM.

**Figure 8:**
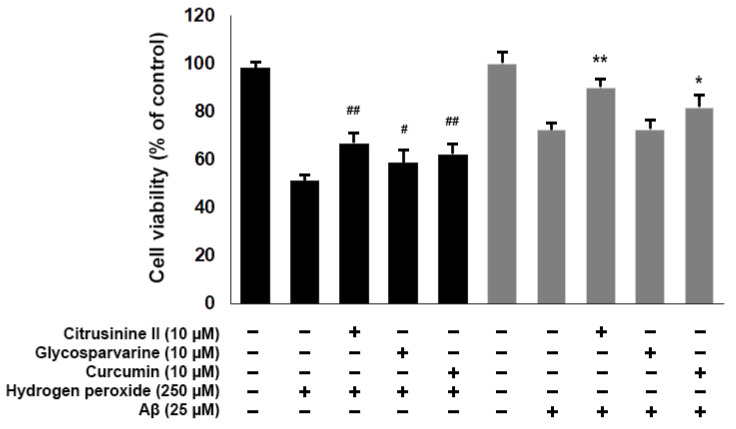
Effects of citrusinine II and glycosparvarine on H_2_O_2_ and Aβ_1–42_ induced cell death in SH-SY5Y cells. Curcumin at 10 µM was used as a reference standard. The values are reported as mean ± SD (*n* = 4). One-way ANOVA followed by the Tukey test, # *p* < 0.05, ## *p* < 0.01 versus H_2_O_2_ treated group, * *p* < 0.05, ** *p* < 0.01 versus Aβ_1–42_ treated group.

To examine the neuroprotective effects of acridones against H_2_O_2_, SH-SY5Y cells were pre-treated with 10 µM citrusinine II and glycosparvarine for 24 h before exposure to 250 µM H_2_O_2_ for 2 h. The treatment of cells with 250 µM H_2_O_2_ alone resulted in an approximately 51% loss of cell viability compared to control. The cytotoxic effects of H_2_O_2_ were attenuated by pre-treating cells with citrusinine II and glycosparvarine (Figure 8). For evaluation of the neuroprotective effects of acridones against Aβ_1-42_ toxicity, SH-SY5Y cells were pre-treated with 10 µM citrusinine II and glycosparvarine for 24 h, followed by 25 µM Aβ_1-42_ for 24 h. Cell viability was determined by MTT assay. Only citrusinine II significantly protected the SH-SY5Y cells from Aβ_1-42_ toxicity, as shown in Figure 8. Interestingly, citrusinine II exhibited better neuroprotection than curcumin, the reference standard. Taken together, these findings suggested that citrusinine II could protect neuronal cell death by both H_2_O_2_ and Aβ_1-42_.

## 3. Materials and Methods

### 3.1. Acridones and Materials

Ten acridones were extracted from the stems of *Atalantia monophylla*, including *N*-methylatalaphylline, atalaphylline, *N*-methylatalaphyllinine, atalaphyllinine, *N*-methylcycloatalaphylline A, citrusinine I, citrusinine II, glycosparvarine, citruscridone, and buxifoliadine C (Figure 1). The isolation and structural elucidation is described elsewhere [45]. Before use, all acridones were prepared as stock solution in ethanol or DMSO at a concentration of 10 mM. Acetylthiocholine iodide (ATCI), beta amyloid 1-42 (Aβ_1-42_), tacrine, trolox, trypsin, fetal bovine serum (FBS), and Dulbecco’s modified Eagle medium nutrient mixture F-12 (DMEM/F12) were purchased from Sigma-Aldrich (SM Chemical supplies Co., Ltd., Bangkok, Thailand), Merck (Merck, Bangkok, Thailand), Gibthai (GT Chemical supplies Co., Ltd., Bangkok, Thailand), and Fluka (SM Chemical supplies Co., Ltd., Bangkok, Thailand). Human neuroblastoma cell line SH-SY5Y cells were purchased from ATCC (A.N.H. Scientific Marketing Co., Ltd., Bangkok, Thailand). AutoDock program version 4.2.6 (the Scripps Research Institute, La Jolla, CA, USA) and BIOVIA Discovery Studio 2017 (BIOVIA, San Diego, CA, USA) were used in the molecular docking study.

### 3.2. In Silico Physicochemical Properties

SwissADME webserver [59] was used to assess the physicochemical properties for determination of the good drug candidates. In this study, the physicochemical parameters (molecular weight, topological polar surface area (TPSA), number of rotatable bonds, number of H-bond acceptors, and number of H-bond donors), and lipophilicity were checked for all test compounds. Together, the Lipinski and Veber’s rules were used to verify the drug-likeness profile, and all investigated compounds did not violate these rules.

### 3.3. In Vitro ABTS^•+^ Scavenging Activity

The antioxidant activity was determined by the decolonization of the radical cation of 2,2′-azinobis-(3-ethylbenzothiazoline-6-sulfonic acid) (ABTS^•+^) [60]. The ABTS^•+^ was generated by the reaction between 7 mM ABTS and 2.45 mM potassium persulfate (K_2_S_2_O_8_) in water in the dark at room temperature for 12–16 h. Prior to the assay, the ABTS^•+^ solution was diluted to get an absorbance of 0.70 ± 0.02 at 700 nm with ethanol. Trolox was used as an antioxidant standard. All test and standard compounds were dissolved in ethanol. Fifty microliters of each test compound was added to 100 µL of diluted ABTS^•+^ solution to obtain final concentrations from 10 to 100 µM. The results are represented as IC_50_, which is the test compound concentration providing 50% inhibition of free radicals. Experiments were performed in independent triplicates.

### 3.4. In Vitro AChE Inhibitory Activity

AChE activity was evaluated and modified from Ellman’s method [61]. All test compounds were prepared as stock solution in ethanol. The enzyme activity was measured in a mixture of phosphate buffer (0.1 M, pH 7.4), acetylthiocholine (1 mM), 5,5-dithio-bis-(2-nitrobenzoic acid) (DTNB, Ellman’s reagent, (1 mM)), 0.2 Units/mL AChE from *Electrophorus electricus* (type VI-S), and various concentrations of test compounds in which the final concentration ranged from 10 to 100 µM. Tacrine was used as a reference standard. The absorbance was measured by UV spectroscopy (METERTECH Accureader M965) at the wavelength of 405 nm for 5 min. The percentage inhibition was calculated and is shown as IC_50_. Experiments were performed in triplicates.

### 3.5. Kinetic Analysis of AChE Inhibition

To study the mechanism of AChE inhibitory action, the kinetic measurements were performed using Ellman’s method. The rate of the enzymatic reaction was obtained with different concentrations of test compounds (1, 10, 50, 100 µM) and in the absence of test compounds against five concentrations of substrate acetylthiocholine (0.1, 0.25, 0.5, 1, 2 mM). The kinetic characterization was recorded at a wavelength of 405 nm for 5 min. The kinetic analysis was performed by using Lineweaver–Burk plots to determine the type of inhibition. All measurements were performed in triplicate and the calculations were performed using an excel spreadsheet.

### 3.6. Inhibition of Aβ_1-42_ Aggregation

The Aβ_1-42_ self-aggregation process was studied using the thioflavin-T assay [62,63]. Aβ_1-42_ monomers from humans (abcam, U.K.) were solubilized with 50 mM phosphate buffer (pH 7.4) to give a 250 µM solution as a stock solution. All test compounds were dissolved by DMSO. Curcumin was used as a reference standard. Five microliters of test compound was added to black, opaque 96-well plates in various concentrations. Then, Aβ_1-42_ solution was added to each well to a final concentration of 10 µM and gently mixed. The final concentrations of test compounds ranged from 1 to 10 µM. Plates were covered and kept in the dark for 48 h at 37 °C with no agitation. After incubation, 175 µL of 5 µM ThT in 50 mM glycine/NaOH buffer (pH 8.0) was added to each well and the fluorescence intensities were measured at excitation and emission wavelengths of 446 nm and 490 nm, respectively. The percent inhibition of the test compounds was calculated and is shown as IC_50_. All experiments were done in triplicate.

### 3.7. Computational Studies

#### 3.7.1. In Silico Binding Interaction Studies between Acridones and Targets

To perform the molecular docking studies, an AChE template was prepared from an X-ray crystal structure (PDB code: 2CEK) and validated by six AChE inhibitors 2CEK, 1H22, 1ZGB, 2CMF, 1ODC, and 1UT6 [64]. All water and other solvent molecules in the templates of AChE were extracted and hydrogens, Gasteiger charges, and merged non-polar hydrogen atoms were added. The grids were designated and generated by AutoGrid to include the active site of AChE. The grid box dimensions were defined with a size of 80 × 70 × 70 and the grid spacing was set to 0.375 Å. The compounds were drawn and energy minimized with ChemDraw and Chem3D 15.1 software. A Lamarckian genetic algorithm protocol was set by using a population size of 100 individuals with 100 ligand orientation runs. Additionally, the energy evaluation was 1,000,000 and the maximum number of evaluations was 27,000. All ligands were docked by using the Lamarckian genetic algorithm via the Autodock 4.2.6 program. The orientation with the lowest docked energy was considered as the best conformation. After the docking process, the docking complex poses were analyzed for their interactions by using BIOVIA Discovery Studio 2017. In the case of the Aβ fibril, the template was prepared using the X-ray crystal PDB code 2BEG. The template and ligand preparation were the same as above. The grid box dimensions were defined with a size of 120 × 60 × 40 Å with 0.375 Å grid spacing. The docking was performed by the Autodock 4.2.6 program and carried out with 100 runs using the Lamarckian genetic algorithm. Furthermore, the energy evaluation and maximum number of evaluations were set to 1,000,000 and 27,000, respectively. The best orientations with the lowest docked energies were visualized for their interactions by using BIOVIA Discovery Studio 2017.

#### 3.7.2. In Silico Drug ADMET Evaluation

pKCSM is an accessible web server that is used to evaluate the pharmacokinetics of small molecules by analyzing parameters of absorption, distribution, metabolism, excretion, and toxicity (ADMET) [55]. The absorption factor depends on intestinal absorption. The brain distribution is assessed by the blood–brain barrier (logBB) permeability. Metabolism is predicted based on the CYP inhibition model (CYP2C9 and CYP3A4). Excretion is predicted based on the total clearance and renal OCT2 substrate. The toxicity of drugs is predicted based on the hepatotoxicity.

### 3.8. Neuroprotective Activity against Hydrogen Peroxide (H_2_O_2_) and Aβ_1-42_ Induced Cell Death in Human Neuroblastoma SH-SY5Y Cells

Human neuroblastoma cell line SH-SY5Y cells were cultured in Dulbecco’s modified Eagle medium (DMEM)/Ham’s F-12 medium supplemented with 10% fetal bovine serum (FBS), 100 U/mL penicillin and 100 µg/mL streptomycin. Cells were routinely cultured in 75 cm^2^ cell culture flasks at 37 °C in 5% CO_2_. After that, cells were seeded at a density 5 × 10^5^ cells/mL in 96-well plates and incubated for an additional 48 h before evaluation.

For the investigation of the toxicity effect of test compounds, cells were treated with test compounds at final concentrations of 0.1, 1, 10, 100 μM for 24 h. All compounds were dissolved in DMSO as a stock solution and the final concentration of DMSO did not exceed 0.1% in culture media. Then, culture media was replaced with a solution of MTT (0.5 mg/mL) at 37 °C for 2 h to determine survival of cells. Live cells containing formazan crystals were solubilized in 100 µL of DMSO. The absorption was measured by UV spectrometer (METERTECH Accureader M965) at 570 nm. The percentages of cell viability were calculated in relation to untreated cells.

For evaluation of the neuroprotective effect against H_2_O_2_ induced cell damage, cells were pretreated with test compounds or a reference standard at the concentration of 10 μM for 24 h. After treatment, the medium was removed, and cells were washed with phosphate buffer saline to remove unabsorbed test compounds. After that, the cells were incubated in medium containing 250 µM of H_2_O_2_ at 37 °C for 2 h. The cell viability was determined by MTT assay. Curcumin at a concentration of 10 µM was used as a reference standard.

For neuroprotection against Aβ_1–42_ toxicity, the Aβ_1–42_ preparation was modified from Thiratmatrakul et al. [65]. Briefly, lyophilized Aβ_1–42_ was reconstituted in sterile water and kept at –80 °C until use. Aliquots were diluted with serum-free medium to achieve a final concentration of 25 μM and then incubated at 37 °C for 72 h to form aggregated amyloid. For assay, SH-SY5Y cells were pretreated with test compounds or a reference standard at the concentration of 10 μM for 24 h. After that, to remove unabsorbed test compounds, the medium was removed, and cells were washed with phosphate buffer saline. Then the cells were incubated with aggregated Aβ (25 µM). Twenty-four hours later, cell viability was determined by the MTT colorimetric method. All experiments were done in independent triplicates (4 wells/group).

### 3.9. Statistical Analysis

For statistical analysis, IBM SPSS Statistics 26.0 for Windows was used. All data is represented as the mean ± standard deviation (SD). One-way ANOVA was performed for all experimental analysis with Tukey post hoc test. Statistical significance was considered for *p* < 0.05 and *p* < 0.01.

## 4. Conclusions

Ten acridones isolated from *Atalantia monophylla*, namely *N*-methylatalaphylline, atalaphylline, *N*-methylatalaphyllinine, atalaphyllinine, *N*-methylcycloatalaphylline A, citrusinine I, citrusinine II, glycosparvarine, citruscridone, and buxifoliadine C, were filtered for their drug-likeness properties and evaluated for their activities in three AD pathogenesis-associated mechanisms including antioxidant activity, AChE inhibition, and prevention of beta-amyloid (Aβ) aggregation. All ten acridones showed favorable drug-likeness and possessed multifunctional activities targeting AChE function, Aβ aggregation and oxidation. Structure–activity relationships were also provided in this study. To clarify the mechanism of action, the types of AChE inhibition were investigated in vitro and binding interactions between acridones and AChE or Aβ_1-42_ peptide were explored in silico. Our results demonstrated that all acridones could inhibit AChE in a mixed type manner and bind to AChE at both the catalytic anionic site and the peripheral anionic site, thereby interfering with the hydrolysis of ACh and Aβ aggregation. In silico data showed that acridones could interfere with Aβ_1-42_ aggregation by interacting at the central hydrophobic core, C-terminal hydrophobic region, and the key residues 41 and 42 of Aβ_1-42_, which are the critical segments accounting for Aβ nucleation and fibrillation. In addition, the ADMET profiles of all acridones were predicted in silico using pKCSM programs. Citrisinine II, which showed the most potent multifunctional action with the best ADMET profile, alleviated neuronal cell damage induced by hydrogen peroxide and Aβ_1-42_ toxicity. Conclusively, all 10 acridones were identified as multi-target agents capable of inhibiting the pathogenesis of AD. The structure–activity relationship information obtained from this study might be applied for further optimization of acridone derivatives into a useful drug for AD treatment. Citrusinine II could serve as a candidate with potential impact for further development in Alzheimer’s treatment. However, the elucidation of the mechanisms of action is an important step in the search for new drug candidates. Thus, further studies focusing on the mechanism of action of the selected compound citrusinine II will be planned in the near future.

## Figures and Tables

**Table 1:** Physicochemical properties of acridones predicted by in silico analysis (SwissADME program).

Compounds	Physicochemical Properties					
	MW (g/mol)	TPSA ^a^ (Å^2^)	Num. Rotatable Bonds	Num. H-Bond Acceptors	Num. H-Bond Donors	Log Po/w ^b^
N-methylatalaphylline	393.48	82.69	4	4	3	4.55
Atalaphylline	379.45	93.55	4	4	4	4.61
N-methylatalaphyllinine	391.46	71.69	2	4	2	4.31
Atalaphyllinine	379.45	82.55	2	4	3	4.51
N-methylcycloatalaphylline A	391.46	71.69	2	4	2	4.37
Citrusinine II	287.27	91.92	1	5	3	1.68
Citrusinine I	301.29	80.92	2	5	2	2.04
Glycosparvarine	287.37	91.92	1	5	3	1.66
Citruscridone	317.29	101.15	2	6	3	1.72
Buxifoliadine C	325.36	82.55	3	4	3	4.55

^a^ TPSA topological polar surface area, ^b^ Log Po/w octanol/water partition coefficient.

**Table 2:** The IC_50_ (µM) of ABTS radical scavenging, AChE, and Aβ aggregation inhibitory activities by acridones.

Compounds	ABTS Scavenging Activity	Anti-AChE	Anti-Aβ Aggregation
*N*-methylatalaphylline	50.73 ± 1.02 ^c,d^	49.08 ± 2.75 ^d,e^	7.32 ± 0.09
Atalaphylline	46.24 ± 2.31 ^c^	58.33 ± 2.99 ^f,g,h^	6.58 ± 1.30
*N*-methylatalaphyllinine	57.53 ± 3.52 ^d,e^	54.04 ± 2.03 ^e,f,g^	6.14 ± 3.85
Atalaphyllinine	52.96 ± 1.83 ^c,d,e^	60.78 ± 4.62 ^g,h^	4.79 ± 0.67
*N*-methylcycloatalaphylline A	79.58 ± 3.26 ^f^	66.62 ± 2.51 ^h^	5.84 ± 0.99
Citrusinine II	19.98 ± 3.89 ^a^	42.56 ± 4.81 ^c,d^	6.17 ± 2.29
Citrusinine I	33.77 ± 0.29 ^b^	35.37 ± 1.61 ^c^	7.05 ± 1.45
Glycosparvarine	22.88 ± 2.24 ^a^	18.61 ± 2.77 ^b^	6.58 ± 2.03
Citruscridone	31.69 ± 0.56 ^b^	46.96 ± 2.40 ^d,e^	8.61 ± 0.75
Buxifoliadine C	21.79 ± 2.71 ^a^	50.81 ± 3.74 ^d,e,f^	8.81 ± 3.62
Tacrine	nd	0.28 ± 0.04 ^a^	nd
Trolox	58.92 ± 2.38 ^e^	nd	nd
Curcumin	nd	nd	4.98 ± 0.72

nd = not detected. ^a,b,c,d,e,f,g,h^ Means in a row with different superscript letter are statistically analyzed by one-way ANOVA (*p* < 0.05).

**Table 3:** ADMET parameters of acridones predicted by in silico analysis (pKCSM program).

Name	Absorption	Distribution	Metabolism		Excretion		Toxicity
	Intestinal Absorption (Human) (%Absorbed)	BBB Permeability (Log BB)	CYP2C9 Inhibitor	CYP3A4 Inhibitor	Total Clearance (Log mL/min/kg)	Renal OCT2 Substrate	Hepatotoxicity
N-methylatalaphylline	96.252	−0.864	Yes	No	0.401	No	Yes
Atalaphylline	85.288	−0.887	Yes	No	0.325	No	Yes
N-methylatalaphyllinine	94.544	0.169	Yes	Yes	0.293	No	Yes
Atalaphyllinine	91.158	−0.734	Yes	Yes	0.155	No	Yes
N-methylcycloatalaphylline A	94.951	0.244	Yes	Yes	0.256	No	Yes
Citrusinine II	89.485	−0.996	No	No	0.309	No	No
Citrusinine I	99.555	−0.197	Yes	No	0.423	No	Yes
Glycosparvarine	79.129	−0.948	No	No	0.316	No	No
Citruscridone	68.876	−1.082	Yes	No	0.198	No	No
Buxifoliadine C	93.124	−0.789	Yes	No	0.402	No	Yes

## Data Availability

Data is contained within the article.

## References

[1] Desikan R.S., Sabuncu M.R., Schmansky N.J., Reuter M., Cabral H.J., Hess C.P., Weiner M.W., Biffi A., Anderson C.D., Rosand J. (2010). Selective Disruption of the Cerebral Neocortex in Alzheimer’s Disease. PLoS ONE.

[2] Du X., Wang X., Geng M. (2018). Alzheimer’s disease hypothesis and related therapies. Transl. Neurodegener..

[3] Mu Y., Gage F.H. (2011). Adult hippocampal neurogenesis and its role in Alzheimer’s disease. Mol. Neurodegener..

[4] Tönnies E., Trushina E. (2017). Oxidative Stress, Synaptic Dysfunction, and Alzheimer’s Disease. J. Alzheimer’s Dis..

[5] Volicer L. (2020). Physiological and pathological functions of beta-amyloid in the brain and alzheimer’s disease: A review. Chin. J. Physiol..

[6] Murphy M.P., Levine H. (2010). Alzheimer’s disease and the amyloid-β peptide. J. Alzheimer’s Dis..

[7] Chen G.F., Xu T.H., Yan Y., Zhou Y.R., Jiang Y., Melcher K., Xu H.E. (2017). Amyloid beta: Structure, biology and structure-based therapeutic development. Acta Pharmacol. Sin..

[8] Eanes E.D., Glenner G.G. (1968). X-ray diffraction studies on amyloid filaments. J. Histochem. Cytochem..

[9] Kirschner D.A., Abraham C., Selkoe D.J. (1986). X-ray diffraction from intraneuronal paired helical filaments and extraneuronal amyloid fibers in Alzheimer disease indicates cross-β conformation. Proc. Natl. Acad. Sci. USA.

[10] Caughey B., Lansbury P.T. (2003). Protofibrils, pores, fibrils, and neurodegeneration: Separating the responsible protein aggregates from the innocent bystanders. Annu. Rev. Neurosci..

[11] Soto P., Griffin M.A., Shea J.E. (2007). New insights into the mechanism of Alzheimer amyloid-β fibrillogenesis inhibition by N-methylated peptides. Biophys. J..

[12] Luhrs T., Ritter C., Adrian M., Riek-Loher D., Bohrmann B., Dobeli H., Schubert D., Riek R. (2005). 3D structure of Alzheimer’s amyloid- (1-42) fibrils. Proc. Natl. Acad. Sci. USA.

[13] Petkova A.T., Buntkowsky G., Dyda F., Leapman R.D., Yau W.M., Tycko R. (2004). Solid state NMR reveals a pH-dependent antiparallel β-sheet registry in fibrils formed by a β-amyloid peptide. J. Mol. Biol..

[14] Petkova A.T., Yau W.M., Tycko R. (2006). Experimental constraints on quaternary structure in Alzheimer’s β-amyloid fibrils. Biochemistry.

[15] Tycko R. (2006). Solid-State NMR as a Probe of Amyloid Structure. Protein Pept. Lett..

[16] Tycko R. (2000). Solid-state NMR as a probe of amyloid fibril structure. Curr. Opin. Chem. Biol..

[17] Tycko R. (2006). Molecular structure of amyloid fibrils: Insights from solid-state NMR. Q. Rev. Biophys..

[18] Kheterpal I., Chen M., Cook K.D., Wetzel R. (2006). Structural Differences in Aβ Amyloid Protofibrils and Fibrils Mapped by Hydrogen Exchange—Mass Spectrometry with On-line Proteolytic Fragmentation. J. Mol. Biol..

[19] Hampel H., Mesulam M.M., Cuello A.C., Farlow M.R., Giacobini E., Grossberg G.T., Khachaturian A.S., Vergallo A., Cavedo E., Snyder P.J. (2018). The cholinergic system in the pathophysiology and treatment of Alzheimer’s disease. Brain.

[20] Castro A., Martinez A. (2001). Peripheral and Dual Binding Site Acetylcholinesterase Inhibitors: Implications in treatment of Alzheimer’s Disease. Mini Rev. Med. Chem..

[21] Tougu V. (2001). Acetylcholinesterase: Mechanism of Catalysis and Inhibition. Curr. Med. Chem. Nerv. Syst. Agents.

[22] Inestrosa N.C., Alvarez A., Pérez C.A., Moreno R.D., Vicente M., Linker C., Casanueva O.I., Soto C., Garrido J. (1996). Acetylcholinesterase accelerates assembly of amyloid-beta-peptides into Alzheimer’s fibrils: Possible role of the peripheral site of the enzyme. Neuron.

[23] Alvarez A., Opazo C., Alarcón R., Garrido J., Inestrosa N.C. (1997). Acetylcholinesterase promotes the aggregation of amyloid-β-peptide fragments by forming a complex with the growing fibrils. J. Mol. Biol..

[24] Carlier P.R., Chow E.S.H., Han Y., Liu J., El Yazal J., Pang Y.P. (1999). Heterodimeric tacrine-based acetylcholinesterase inhibitors: Investigating ligand-peripheral site interactions. J. Med. Chem..

[25] Bartolini M., Bertucci C., Cavrini V., Andrisano V. (2003). β-Amyloid aggregation induced by human acetylcholinesterase: Inhibition studies. Biochem. Pharmacol..

[26] Piazzi L., Rampa A., Bisi A., Gobbi S., Belluti F., Cavalli A., Bartolini M., Andrisano V., Valenti P., Recanatini M. (2003). 3-(4-{[benzyl(methyl)amino]methyl}-phenyl)-6,7-dimethoxy-2H-2-chromenone (AP2238) inhibits both acetylcholinesterase and acetylcholinesterase-induced β-amyloid aggregation: A dual function lead for Alzheimer’s disease therapy. J. Med. Chem..

[27] Christen Y. (2000). Oxidative stress and Alzheimer disease. Am. J. Clin. Nutr..

[28] Praticò D., Uryu K., Leight S., Trojanoswki J.Q., Lee V.M.Y. (2001). Increased lipid peroxidation precedes amyloid plaque formation in an animal model of alzheimer amyloidosis. J. Neurosci..

[29] Federico A., Cardaioli E., Da Pozzo P., Formichi P., Gallus G.N., Radi E. (2012). Mitochondria, oxidative stress and neurodegeneration. J. Neurol. Sci..

[30] Yan M.H., Wang X., Zhu X. (2013). Mitochondrial defects and oxidative stress in Alzheimer disease and Parkinson disease. Free Radic. Biol. Med..

[31] Cassidy L., Fernandez F., Johnson J.B., Naiker M., Owoola A.G., Broszczak D.A. (2020). Oxidative stress in alzheimer’s disease: A review on emergent natural polyphenolic therapeutics. Complement. Ther. Med..

[32] Allan Butterfield D., Castegna A., Lauderback C.M., Drake J. (2002). Evidence that amyloid beta-peptide-induced lipid peroxidation and its sequelae in Alzheimer’s disease brain contribute to neuronal death. Neurobiol. Aging.

[33] Butterfield D.A., Boyd-Kimball D. (2019). Redox proteomics and amyloid β-peptide: Insights into Alzheimer disease. J. Neurochem..

[34] Butterfield D.A. (2002). Amyloid β-peptide (1-42)-induced oxidative stress and neurotoxicity: Implications for neurodegeneration in Alzheimer’s disease brain. A review. Free Radic. Res..

[35] Grundman M., Delaney P. (2002). Antioxidant strategies for Alzheimer’s disease. Proc. Nutr. Soc..

[36] Staehelin H.B. (2005). Micronutrients and Alzheimer’s disease. Proc. Nutr. Soc..

[37] Davis K.L., Powchick P. (1995). Tacrine. Lancet.

[38] Crismon M.L. (1994). Tacrine: First drug approved for alzheimer’s disease. Ann. Pharmacother..

[39] Qizilbash N., Birks J., Lopez A.J., Lewington S., Szeto S. (2007). Tacrine for Alzheimer’s disease (Withdrawn paper. 1999, art. no. CD000202). Cochrane Database Syst. Rev..

[40] Cholewiñski G., Dzierzbicka K., Kolodziejczyk A.M. (2011). Natural and synthetic acridines/acridones as antitumor agents: Their biological activities and methods of synthesis. Pharmacol. Rep..

[41] Wouatsa V.N., Misra L., Kumar S., Prakash O., Khan F., Tchoumbougnang F., Venkatesh R.K. (2013). Aromatase and glycosyl transferase inhibiting acridone alkaloids from fruits of Cameroonian Zanthoxylum species. Chem. Cent. J..

[42] Han Y., Luo A., Kamau P., Takomthong P., Hu J., Boonyarat C., Luo L., Lai R. (2021). A plant-derived TRPV3 inhibitor suppresses pain and itch. Br. J. Pharmacol..

[43] Yamamoto N., Furukawa H., Ito Y., Yoshida S., Maeno K., Nishiyama Y. (1989). Anti-herpesvirus activity of citrusinine-I, a new acridone alkaloid, and related compounds. Antiviral Res..

[44] Parveen M., Aslam A., Nami S.A.A., Malla A.M., Alam M., Lee D.-U., Rehman S., Silva P.S.P., Silva M.R. (2016). Potent acetylcholinesterase inhibitors: Synthesis, biological assay and docking study of nitro acridone derivatives. J. Photochem. Photobiol. B Biol..

[45] Sombatsri A., Thummanant Y., Sribuhom T., Boonmak J., Youngme S., Phusrisom S., Kukongviriyapan V., Yenjai C. (2018). New limonophyllines A-C from the stem of *Atalantia monophylla* and cytotoxicity against cholangiocarcinoma and HepG2 cell lines. Arch. Pharm. Res..

[46] Lipinski C.A., Lombardo F., Dominy B.W., Feeney P.J. (2001). Experimental and computational approaches to estimate solubility and permeability in drug discovery and development settings. Adv. Drug Deliv. Rev..

[47] Veber D.F., Johnson S.R., Cheng H.Y., Smith B.R., Ward K.W., Kopple K.D. (2002). Molecular properties that influence the oral bioavailability of drug candidates. J. Med. Chem..

[48] Vajragupta O., Toasaksiri S., Boonyarat C., Wongkrajang Y., Peungvicha P., Watanabe H., Boonchoong P. (2000). Chroman amide and nicotinyl amide derivatives: Inhibition of lipid peroxidation and protection against head trauma. Free Radic. Res..

[49] Blokland A., Geraerts E., Been M. (2004). A detailed analysis of rats’ spatial memory in a probe trial of a Morris task. Behav. Brain Res..

[50] Hasselmo M.E. (2006). The role of acetylcholine in learning and memory. Curr. Opin. Neurobiol..

[51] Ahmed M., Davis J., Aucoin D., Sato T., Ahuja S., Aimoto S., Elliott J.I., Van Nostrand W.E., Smith S.O. (2010). Structural conversion of neurotoxic amyloid-Β 1-42 oligomers to fibrils. Nat. Struct. Mol. Biol..

[52] Antzutkin O.N., Leapman R.D., Balbach J.J., Tycko R. (2002). Supramolecular structural constraints on Alzheimer’s β-amyloid fibrils from electron microscopy and solid-state nuclear magnetic resonance. Biochemistry.

[53] Marina G.B., Kirkitadze D., Lomakin A., Vollers S.S., Benedek G.B., Teplow D.B. (2003). Amyloid β-protein (Aβ) assembly: Aβ40 and Aβ42 oligomerize through distinct pathways. Proc. Natl. Acad. Sci. USA.

[54] Urbanc B., Cruz L., Yun S., Buldyrev S.V., Bitan G., Teplow D.B., Stanley H.E. (2004). In silico study of amyloid β-protein folding and oligomerization. Proc. Natl. Acad. Sci. USA.

[55] Pires D.E.V., Blundell T.L., Ascher D.B. (2015). pkCSM: Predicting small-molecule pharmacokinetic and toxicity properties using graph-based signatures. J. Med. Chem..

[56] Ohashi R., Watanabe R., Esaki T., Taniguchi T., Torimoto-Katori N., Watanabe T., Ogasawara Y., Takahashi T., Tsukimoto M., Mizuguchi K. (2019). Development of Simplified in Vitro P-Glycoprotein Substrate Assay and in Silico Prediction Models to Evaluate Transport Potential of P-Glycoprotein. Mol. Pharm..

[57] Furge L.L., Guengerich F.P. (2006). Cytochrome P450 enzymes in drug metabolism and chemical toxicology: An introduction. Biochem. Mol. Biol. Educ..

[58] Tumiatti V., Minarini A., Bolognesi M.L., Milelli A., Rosini M., Melchiorre C. (2010). Tacrine Derivatives and Alzheimers Disease. Curr. Med. Chem..

[59] Daina A., Michielin O., Zoete V. (2017). SwissADME: A free web tool to evaluate pharmacokinetics, drug-likeness and medicinal chemistry friendliness of small molecules. Sci. Rep..

[60] Chheng C., Waiwut P., Plekratoke K., Chulikhit Y., Daodee S., Monthakantirat O., Pitiporn S., Musigavong N., Kwankhao P., Boonyarat C. (2020). Multitarget activities of kleeb bua daeng, a Thai traditional herbal formula, against alzheimer’s disease. Pharmaceuticals.

[61] Ellman G.L., Courtney K.D., Andres V., Featherstone R.M. (1961). A new and rapid colorimetric determination of acetylcholinesterase activity. Biochem. Pharmacol..

[62] LeVine H. (1999). Quantification of beta-sheet amyloid fibril structures with thioflavin T. Methods Enzymol..

[63] Khamphukdee C., Monthakantirat O., Chulikhit Y., Buttachon S., Lee M., Silva A.M.S., Sekeroglu N., Kijjoa A. (2018). Chemical Constituents and Antidepressant-Like Effects in Ovariectomized Mice of the Ethanol Extract of Alternanthera philoxeroides. Molecules.

[64] Takomthong P., Waiwut P., Yenjai C., Sripanidkulchai B., Reubroycharoen P., Lai R., Kamau P., Boonyarat C. (2020). Structure-activity analysis and molecular docking studies of coumarins from toddalia asiatica as multifunctional agents for alzheimer’s disease. Biomedicines.

[65] Thiratmatrakul S., Yenjai C., Waiwut P., Vajragupta O., Reubroycharoen P., Tohda M., Boonyarat C. (2014). Synthesis, biological evaluation and molecular modeling study of novel tacrine-carbazole hybrids as potential multifunctional agents for the treatment of Alzheimer’s disease. Eur. J. Med. Chem..

